# Unveiling Sex Differences in Tricuspid Valve Disease: A Systematic Review and Meta-Analysis of Surgical Management

**DOI:** 10.7759/cureus.50478

**Published:** 2023-12-13

**Authors:** Ayesha Islam Khan, Tahoora N Surve, Virushnee Senthilkumar, Nishant Kumar, Abdul Haseeb, Shinto Bosco, Soujanya Tirupati, Rajeswari Ramalingame, Asem M Thaher, Saya Alasaadi, Umer Suleman

**Affiliations:** 1 Department of Medicine, Allama Iqbal Medical College, Lahore, PAK; 2 Department of Medicine, K. J. Somaiya Medical College and Research Centre, Mumbai, IND; 3 Department of Surgery, Coimbatore Medical College and Hospital, Salem, IND; 4 Department of Surgery, Christian Medical College and Hospital, Vellore, IND; 5 Department of Medicine, District Health Office, Palandri, PAK; 6 Department of Anesthesia, Postgraduate Institute of Medical Education and Research, Chandigarh, IND; 7 Department of Anesthesia, MediCiti Institute of Medical Sciences, Medchal, IND; 8 Department of Medicine, Aarupadai Veedu Medical College, Puducherry, IND; 9 Department of Medicine, California Institute of Behavioral Neurosciences and Psychology, Fairfield, USA; 10 Department of Medicine, University College Dublin, Dublin, IRL; 11 Department of Medicine, Jamaica Hospital Medical Center, New York, USA

**Keywords:** permanent pacemaker, mortality, treatment outcomes, tricuspid valve repair, open heart surgery, sex differences, tricuspid regurgitation, transcatheter tricuspid valve replacement

## Abstract

Tricuspid regurgitation (TR) is a heart condition where blood flows backward through the tricuspid valve. Tricuspid valve disease constitutes a major valvular heart condition that is receiving heightened attention due to tailored treatment options and sex-specific differences in treatment outcomes. The study aims to investigate whether biological sex has a significant influence on the development, progression, and treatment outcomes of tricuspid valve disease in adults. We conducted a comprehensive search to identify studies examining the impact of sex on the pathophysiology of TR as well as treatment outcomes in patients with TR. We searched PUBMED/MEDLINE, SCOPUS, and Excerpta Medica dataBASE (EMBASE) from inception to September 2023 to identify relevant studies. Twelve studies totaling 22,574 patients met our eligibility criteria. These studies were categorized into three subgroups: patients with TR without intervention (3,848 patients, with 48.1% males and 51.9% females), those who underwent open heart surgery (17,498 patients: 46.2% males and 53.8% females), and those who underwent transcatheter tricuspid valve repair/replacement (TTVR; 1,687 patients: 41.6% males and 58.4% females). Analysis revealed no major differences in terms of TR etiology. Males tended to have a slightly lower mean age difference (mean difference (MD): -0.60 years; 95% confidence interval (CI) (-1.49, -0.04); p = 0.10) but had more frequent chronic lung disease (risk ratio (RR): 1.12, 95% CI (1.01, 1.25), p = 0.03). Males showed higher baseline TR volume (MD: 4.11, 95% CI (0.53, 7.68), p = 0.02) and lower left ventricular ejection fraction (MD: -5.85, 95% CI (-6.97, -4.73), p < 0.00001). Following open heart surgery for TR treatment, males required more frequent permanent pacemaker implantation (PPM; RR: 1.57, 95% CI (1.21, 2.03), p = 0.0006). Similarly, TTVR showed a higher need for PPM in males (RR: 1.45, 95% CI (1.10, 1.93), p = 0.010). In-hospital mortality exhibited no sex differences, but males had a slightly elevated late mortality risk. Sex differences in TR patients were notable in baseline characteristics, with males having a higher risk of certain conditions. The more frequent requirement for PPM was a major sex-based difference in terms of prognosis.

## Introduction and background

Tricuspid regurgitation (TR), also known as tricuspid insufficiency, is a heart condition where blood flows backward through the tricuspid valve [1]. It can be categorized as primary, caused by intrinsic valve abnormalities, or secondary, often due to right ventricular or tricuspid annular dilation [2]. Causes of primary TR, which is less common in adults, include valve injury from medical procedures, trauma, infective endocarditis, and congenital conditions [3]. On the contrary, secondary TR is more prevalent and results from right ventricular dilation and tricuspid annular dilation with a previously normal valve [4].

Despite around 1.6 million Americans having moderate to severe TR, only a few thousand repair procedures are performed yearly [5]. Neglected severe TR can lead to poor outcomes, emphasizing the need for more attention to this condition [6].

Patients with TR may experience symptoms of right-sided heart failure, and diagnosis is primarily done through Doppler echocardiography, which reveals tricuspid valve motion abnormalities and right heart chamber dilation [1,7]. Treatment depends on severity and underlying causes, including pharmacotherapy and surgery [1]. Surgery is considered for patients needing left-sided valve surgery or those with severe TR and right ventricular dysfunction [8].

Surgical repair of the tricuspid valve is a common approach for managing TR. In cases of secondary TR, repair is typically considered. Primary TR, involving complex valve leaflet pathology, may necessitate valve replacement for better outcomes [9].

Surgical techniques for tricuspid valve repair include suture tricuspid valve annuloplasty, ring annuloplasty, and the clover technique [10,11,12]. In recent years, transcatheter tricuspid valve repair/replacement (TTVR) has emerged as a promising treatment option. These procedures address issues like leaflet coaptation, annular dilation, and prosthetic valve deployment [13]. Three-dimensional transesophageal echocardiography (TEE) is crucial for guiding the precise placement of clips during the procedure.

While a focus is observed on enhancing the management and outcomes of TR, sex-specific differences in TR treatment are an important area for further exploration, as existing studies lack such data. Analyzing these differences can help tailor treatment approaches to individual patient needs.

Sex differences in biology, including body size, cardiac anatomy, and hormones, can impact treatment outcomes. However, sex disparities in tricuspid valve surgery research remain limited. In our systematic review and meta-analysis, we aimed to collate evidence on sex-specific differences in terms of disease characteristics and management outcomes.

## Review

Materials and methods

Literature Searches

**Figure 1:**
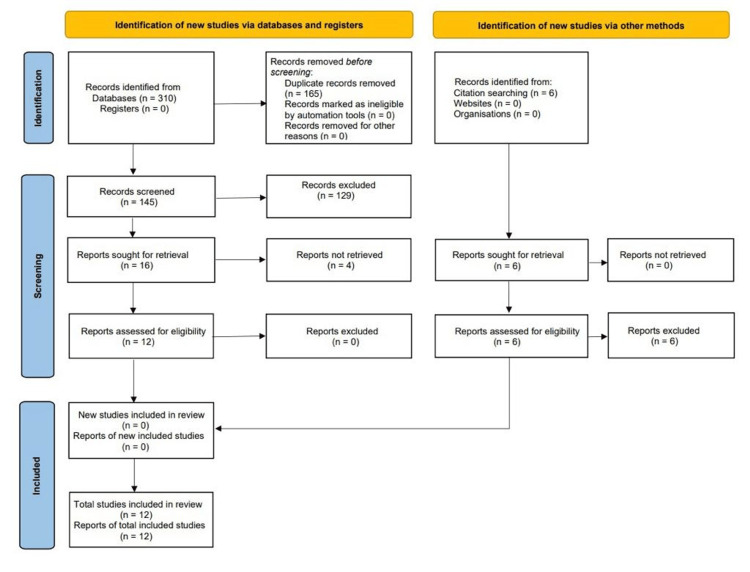
PRISMA chart The flowchart represents the methodology of the article on sex differences in tricuspid valve disease

The Population, Intervention, Comparator, and Outcome (PICO) standard was used in the development of search terms to detect studies that provide comparisons of sex-specific treatment outcome comparisons in adult patients who have undergone tricuspid valve surgery. Our search covered several databases, including PUBMED/MEDLINE, SCOPUS, and Excerpta Medica dataBASE (EMBASE), up to September 2023. To guarantee a robust search strategy, we followed the Preferred Reporting Items for Systematic Reviews and Meta-Analyses (PRISMA) statement and adhered to the prescribed guidelines (Figure 1) [14,15].

The search terms employed were "sex" or "gender" and "tricuspid regurgitation" or "tricuspid insufficiency” or “tricuspid valve disease” or “TR” and "transcatheter tricuspid valve repair" or "TTVR" or "tricuspid surgery" or "tricuspid valve replacement."

To ensure that we did not miss any relevant studies, we conducted a thorough backward-forward citation check. The ultimate choice of studies was made according to predetermined inclusion and exclusion criteria.

Inclusion and Exclusion Criteria

Each paper was carefully assessed during the abstract and full-text review stages by two impartial reviewers, with a third reviewer resolving any discordance.

Inclusion criteria: (1) The research must provide a minimum of one comparative sex-based outcome following tricuspid valve surgery. (2) The number of subjects participating in each group (male and female) should be stated. (3) The language in which the study is published should be English. (4) Only studies with human subjects were taken into consideration. (5) Every participant in the study must be an adult (aged >18 years). (6) Clinical trials or observational cohort studies (both prospective and retrospective) were study designs that were included. (7) Abstracts are added if they comply with all the conditions mentioned above and are the sole source of available information.

Exclusion criteria: (1) Research that is published in languages other than English. (2) Studies that state interventions or treatments other than tricuspid valve surgery. (3) Studies that do not provide treatment outcomes stratified by sex. (4) Study designs that do not satisfy the inclusion criteria, comprising case reports/series, narrative reviews, editorials, systematic reviews, meta-analyses, study protocols, and abstracts.

Data Extraction and Quality Assessment

Five independent investigators were tasked with data extraction from the included studies, and a thorough review of the data was performed. A single impartial reviewer resolved any discrepancies that emerged during this process so that the accuracy of the data and consistency were guaranteed. The event and total numbers were extracted for each group, and continuous data was represented with mean and standard deviations for categorical data. Data that only had a median was converted to a mean using the formula created by Wan et al. [16]. Thirty days was the minimum follow-up period for short-term evaluations and a minimum of three months for mid-term follow-up to assess the treatment outcomes.

The extracted data primarily focused on key demographic characteristics, including age, sex, and baseline factors like pulmonary artery systolic pressure (PASP), left ventricular ejection fraction (LVEF), tricuspid annular plane systolic excursion (TAPSE), TR severity, history of chronic lung disease, concurrent mitral regurgitation, and their relationship with sex's impact on tricuspid valve surgery outcomes. Additionally, the number of subjects with a baseline functional classification greater than three, according to the New York Heart Association, was considered.

The primary outcomes of interest included in-hospital mortality, late mortality, the development of new arrhythmias, the need for a PPM, and the occurrence of acute kidney injury (AKI). Using the Newcastle-Ottawa Scale (NOS) risk-of-bias assessment instrument, the studies that met the inclusion criteria were assessed. After two impartial assessors evaluated the potential for bias, a final table was created based on their consensus (Table 1).

**Table 1:** Risk-of-bias assessment using the NOS Scotti et al. 2022 [17]; Veen et al. 2018 [18]; Chandrashekar et al. 2018 [19]; Dietz et al. 2021 [20]; Gual-Capllonch et al. 2020 [21]; Prihadi et al. 2018 [22]; Hui et al. [23]; Khan et al. 2023 [24]; Pfannmueller et al. 2012 [25]; Fortmeier et al. 2023 [26]; Leviner et al. 2014 [27]; Saeed et al. 2021 [28] ^# ^Follow-up length was determined to be 30 days for the short-term and at least three months for the mid-term. Adequacy of follow-up meant less than 10% loss at 30 days NOS: Newcastle-Ottawa scale

NOS		Reference											
		[17]	[18]	[19]	[20]	[21]	[22]	[23]	[24]	[25]	[26]	[27]	[28]
Selection	Representativeness of the exposed	★	★	★	★	★	★	★	★	★	★	★	★
	Selection of non-exposed	★	★	★	★	★	★	★	★	★	★	★	★
	Ascertainment of exposure	★	★	★	★	★	★	★	★	★	★	★	★
	Outcome of interest not present at study beginning	★	★	★	★	★	★	★	★	★	★	★	★
Comparability	Main factor	★	★	★	★	★	★	★	★	★	★	★	★
	Additional factor	★	0	★	★	★	0	0	0	0	★	★	0
Outcome	Assessment	★	★	★	★	★	★	★	★	★	★	★	★
	Follow-up length^#^	★	★	★	★	★	★	★	★	★	★	★	★
	Adequacy of follow-up	★	★	★	★	★	★	★	★	★	★	★	★
Score		9	8	9	9	9	8	8	8	8	9	9	8

Statistical Analysis

The Cochrane Collaboration and the Meta-analysis of Observational Studies in Epidemiology (MOOSE) guidelines were fully adhered to in this meta-analysis [29]. We used the Cochrane Collaboration’s "Review Manager" software version 5.4.1 (The Cochrane Collaboration, London, UK) for data analysis. We employed the inverse variance random-effects model to estimate the risk ratio (RR) and the associated 95% confidence intervals (CIs) for binary outcomes. The inverse variance method was also utilized to determine the weighted mean difference (MD) for continuous outcomes.

We applied the I² statistics and Q-test for heterogeneity (Cochrane, 1954) to estimate statistical heterogeneity; an I² value greater than 50% denotes substantial heterogeneity. Statistical significance was defined as a p-value less than 0.05.

Results

Demographic Characteristics

In this systematic review and meta-analysis, 12 studies involving 22,574 patients were included to investigate sex-based differences in TR (Table 2). The patient distribution showed 47.5% males and 52.5% females.

**Table 2:** Summary of characteristics of all the included studies in the systematic review TR: tricuspid regurgitation; TVR: tricuspid valve regurgitation; TTVR: transcatheter tricuspid valve repair/replacement

Author	Procedure	Location	Study duration	Study design	Total no. of participants	
					Male	Female
Dietz et al. 2021 [20]	Sex differences in TR	Netherland	1995-2016	Retrospective cohort study	771	798
Gual-Capllonch et al. 2020 [21]		Spain	-		118	133
Prihadi et al. 2018 [22]		Netherland, Belgium	2018		481	465
Hui et al. [23]		China	2014-2015	Prospective cohort study	392	481
Saeed et al. 2021 [28]		Norway, London	Jan 2010-Dec 2010	Retrospective cohort study	92	117
Veen et al. 2018 [18]	Open TVR surgery	Netherland	2007-2016		3080	3141
Chandrashekar et al. 2018 [19]		USA	2004-2013		442	599
Pfannmueller et al. 2012 [25]		Germany	1997-2010		37	55
Leviner et al. 2014 [27]		-	2005-2012	Prospective cohort study	21	46
Fortmeier et al. 2023 [26]	TTVR	Germany	2016-2021		316	386
Scotti et al. 2022 [17]		Europe, America	2016-2021	Retrospective cohort study	240	316
Khan et al. 2023 [24]	Open TVR surgery and TTVR	USA	2015-2019		4660	5846

**Figure 2:**
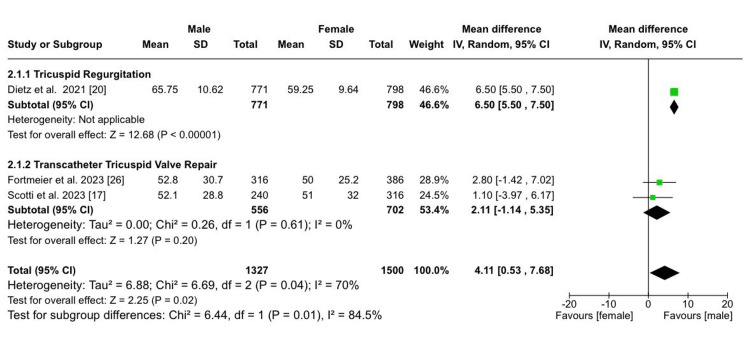
Tricuspid regurgitant volume Sources: References [17,20,26]

**Figure 3:**
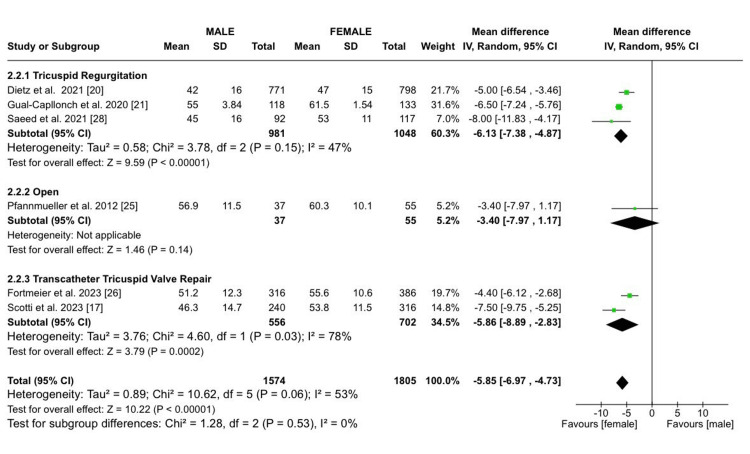
LVEF Sources: References [17,20,21,25,26,28]

These studies were categorized into three subgroups: patients with TR (totaling 3,848 patients, with 48.1% males and 51.9% females), those who underwent open heart surgery (with a total of 17,498 patients, comprising 46.2% males and 53.8% females), and those who underwent TTVR, amounting to 1,687 patients, among which 41.6% were males and 58.4% were females. Demographic analysis revealed that males had a slightly lower mean age (MD: -0.60 years; 95% CI (-1.30, 0.11); p = 0.10; I² = 99%) but more frequent chronic lung disease (RR: 1.12; 95% CI (1.01, 1.25); p = 0.03; I² = 0%). Similar frequencies of concomitant mitral regurgitation were noted between both sexes (RR: 1.05; 95% CI (0.93, 1.18); p = 0.44; I² = 0%). Males also had a significantly higher baseline tricuspid regurgitant volume (MD: 4.11; 95% CI (0.53, 7.68); p = 0.02; I² = 70%; Figure 2), a lower baseline LVEF (MD: -5.85; 95% CI (-6.97, -4.73); p < 0.00001; I²​​​​​​​ = 53%; Figure 3), and a lower baseline TAPSE (MD: -0.59; 95% CI (-1.06, -0.12); p = 0.01; I²​​​​​​​ = 40%) compared to females. Males had a slightly lower baseline PASP; however, the results were not statistically significant (MD: -0.85; 95% CI (-1.91, 0.22); p = 0.12; I²​​​​​​​ = 77%). Table 3 shows the additional details.

**Table 3:** Meta-analysis of baseline demographic characteristics of the study participants * p-value < 0.05 is considered statistically significant. Effect estimate: risk ratio (RR) calculated using inverse variance (IV), random effects model for binary variables #^ ^Effect estimate: mean difference (MD), calculated using inverse variance (IV), random effects model for continuous variables TR: tricuspid regurgitation; TVR: tricuspid valve regurgitation; TTVR: transcatheter tricuspid valve repair/replacement; CI: confidence interval; PASP: pulmonary arterial systolic pressure; SPAP: systolic pulmonary arterial pressure; PPM: permanent pacemaker; LVEF: left ventricular ejection fraction; TAPSE: Tricuspid Annular Plane Systolic Excursion

Baseline characteristics	MD/RR	95% CI	p-value
Sex differences in TR			
Age (years), MD (IV, random, 95% CI)	-0.89^#^	-3.55, 1.78	p = 0.51
PASP or SPAP (mm Hg), MD (IV, random, 95% CI)	0.47^#^	-0.92, 1.87	p = 0.51
LVEF (%), MD (IV, random, 95% CI)	-6.13^#^	-7.38, -4.87	p < 0.00001*
TAPSE (mm), MD (IV, Random, 95% CI)	-0.55^#^	-1.57, 0.47	p = 0.29
Hx of chronic lung disease (n), RR (IV, random, 95% CI)	1.19^#^	0.60, 2.33	p = 0.62
Concomitant mitral regurge (n), RR (IV, random, 95% CI)	1.02^#^	0.89, 1.17	p = 0.79
TR regurgitant volume (mL), MD (IV, random, 95% CI)	6.50^#^	5.50, 7.50	p < 0.00001*
Open TVR surgery group			
Age (years), MD (IV, random, 95% CI)	-0.96^#^	-2.08, 0.15	p = 0.09
LVEF (%), MD (IV, random, 95% CI)	-3.40^#^	-7.97, 1.17	p = 0.14
Hx of chronic lung disease (n), RR (IV, random, 95% CI)	1.08^#^	0.95, 1.21	p = 0.24
TTVR group			
Age (years), MD (IV, random, 95% CI)	-0.01^#^	-0.25, 0.23	p = 0.91
PASP or SPAP (mm Hg), MD (IV, random, 95% CI)	-2.69^#^	-4.34, -1.04	p = 0.001*
LVEF (%), MD (IV, random, 95% CI)	-5.86^#^	-8.89, -2.83	p
TAPSE (mm), MD (IV, random, 95% CI)	-0.50^#^	-1.02, 0.02	p = 0.06
Hx of chronic lung disease (n), RR (IV, random, 95% CI)	1.29^#^	1.03, 1.60	p = 0.02*
Concomitant mitral regurge (n), RR (IV, random, 95% CI)	1.14^#^	0.90, 1.45	p = 0.28
TR regurgitant volume (mL), MD (IV, random, 95 % CI)	2.11^#^	-1.14, 5.35	p = 0.20
Overall			
Age (years), MD (IV, random, 95% CI)	-0.60^#^	-1.30, 0.11	p = 0.10
PASP or SPAP (mm Hg), MD (IV, Random, 95% CI)	-0.85^#^	-1.91, 0.22	p = 0.12
LVEF (%), MD (IV, random, 95% CI)	-5.85^#^	-6.97, -4.73	p < 0.00001*
TAPSE (mm), MD (IV, random, 95% CI)	-0.59^#^	-1.06. -0.12	p = 0.01*
Hx of chronic lung disease (n), RR (IV, random, 95% CI)	1.12^#^	1.01, 1.25	p = 0.03*
Concomitant mitral regurge (n), RR (IV, random, 95% CI)	1.05^#^	0.93, 1.18	p = 0.44
TR regurgitant volume (mL), MD (IV, random, 95 % CI)	4.11^#^	0.53, 7.68	p = 0.02*

Sex-Specific Differences in Treatment Outcomes

Sex-specific differences in treatment outcomes after open heart surgery: The analysis of TR treatment outcomes revealed that there was no statistically significant difference between males and females in both in-hospital mortality (RR: 1.19; 95% CI (0.86, 1.64); p = 0.29; I²​​​​​​​ = 45%) and late mortality (RR: 0.99; 95% CI (0.17, 5.65); p = 0.99) among patients who underwent open heart surgery. In one of the studies, it is highlighted that there were no significant differences in hospital mortality between males and females undergoing isolated tricuspid valve surgery, tricuspid valve repair, and replacement [18]. Similar results were present with no difference in in-hospital mortality/30-day mortality between both sexes. However, they noted that males incurred higher hospital charges, revealing potential sex-based disparities in healthcare costs [19,25]. However, a statistically significant difference emerged between males and females who underwent open heart surgery when the outcome in question was the need for new PPM implantation (RR: 1.57; 95% CI (1.21, 2.03); p = 0.0006), with males showing an increased risk.

Sex-specific differences in treatment outcomes after TTVR: The analysis of TR treatment outcomes showed that there was no statistically significant difference between males and females in both in-hospital mortality (RR: 0.92; 95% CI (0.43, 1.98); p = 0.83; I²​​​​​​​ = 1%) and late mortality (RR: 1.14; 95% CI (0.48, 2.70); p = 0.77) among patients who underwent TTVR. There were no significant sex-based differences in all-cause mortality at one year [17]. Similarly, according to the Kaplan-Meier analysis, there was no sex-based difference in two-year survival rates [26].

However, a statistically significant difference was observed between males and females who underwent TTVR when the outcome of interest was the need for a new PPM (RR: 1.45; 95% CI (1.10, 1.93); p = 0.010), with males having an increased risk. Additionally, it was found that males had a lower risk of developing arrhythmias following TTVR compared to females (RR: 0.26; 95% CI (0.03, 2.24); p = 0.22); however, these results were statistically insignificant [17].

Sex-specific differences in treatment outcomes among patients: In patients who received no medical intervention, the analysis of treatment outcomes revealed a statistically significant higher late mortality in male patients (RR: 1.15; 95% CI (1.03, 1.29); p = 0.02; I²​​​​​​​ = 20%). There was no significant difference in terms of early mortality [28], the development of new arrhythmias (RR: 1.03; 95% CI (0.90, 1.17); p = 0.69; I²​​​​​​​ = 0%), and the need for a new PPM (RR: 1.23; 95% CI (0.82, 1.84); p = 0.31). However, Dietz et al. showed that females have higher one-year, five-year, and 10-year survival rates [20].

**Figure 4:**
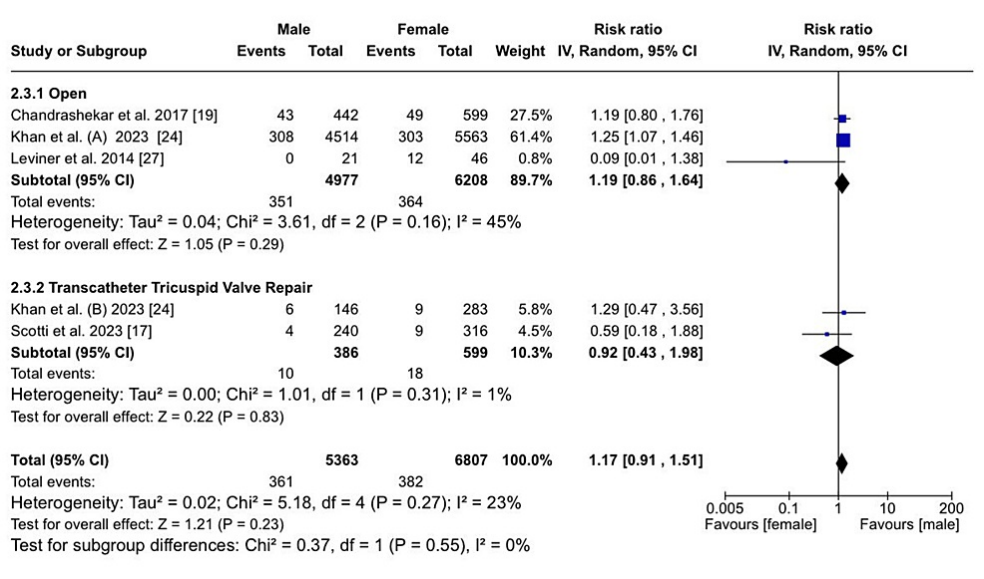
In-hospital mortality Sources: References [17,19,24,27]

Overall sex-specific differences irrespective of treatment option or no treatment: Overall, in-hospital mortality following either open surgery or TTVR was found to be similar between both sexes (RR: 1.17; 95% CI (0.91, 1.51); p = 0.23; I²​​​​​​​ = 23%) (Figure 4).

**Figure 5:**
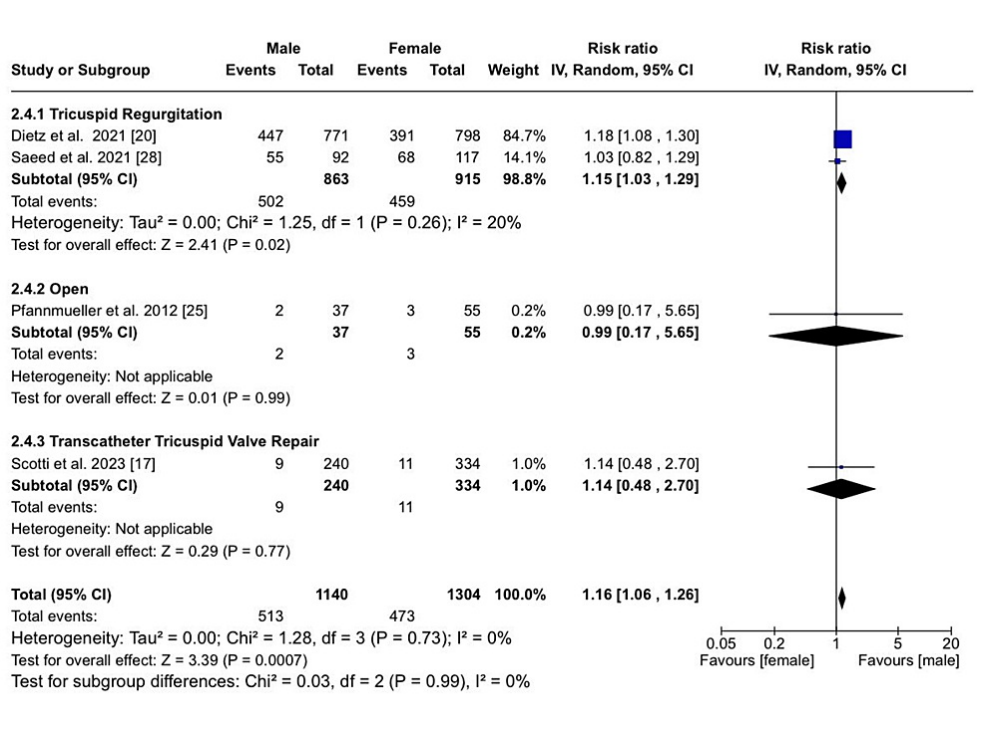
Late mortality Sources: References [17,20,25,28]

However, a statistically significant difference emerged between males and females in terms of late mortality, with males showing a slightly higher risk than females, irrespective of the type of procedure or no procedure (RR: 1.16; 95% CI (1.06, 1.26); p = 0.0007; I²​​​​​​​ = 0%) (Figure 5).

**Figure 6:**
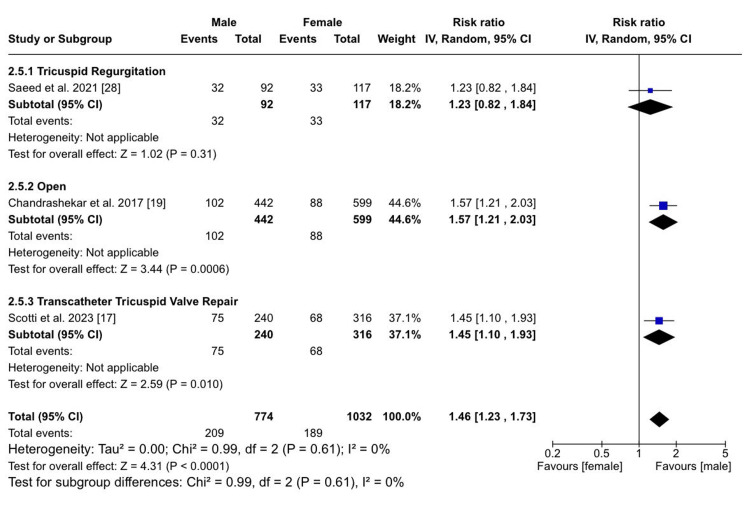
Requirement of PPM implantation Sources: References [17,19,28]

Among other notable outcomes, a statistically significant difference was observed between males and females, with males having an increased risk of the need for a new PPM (RR: 1.46; 95% CI (1.23, 1.73); p < 0.0001; I²​​​​​​​= 0%) (Figure 6).

In terms of developing arrhythmias, males had an overall higher risk with or without any intervention (RR: 1.02; 95% CI (0.90, 1.17); p = 0.75; I²​​​​​​​ = 0%); however, these results were statistically insignificant. Table 4 shows additional details.

**Table 4:** Meta-analysis of sex-based differences in outcomes * p-value < 0.05 is considered statistically significant # Effect estimate: risk ratio (RR), calculated using inverse variance (IV), random effects model for continuous variables TR: tricuspid regurgitation; TVR: tricuspid valve regurgitation; TTVR: transcatheter tricuspid valve repair/replacement; CI: confidence interval; PPM: permanent pacemaker

Outcomes	Effect estimate	95% CI	p-value
In-hospital mortality, RR (IV, random, 95% CI)			
Open TVR surgery	1.19^ǂ^	0.86, 1.64	p = 0.29
TTVR	0.92^ǂ^	0.43, 1.98	p = 0.83
Overall	1.17^ǂ^	0.91, 1.51	p = 0.23
Late mortality, RR (IV, random, 95% CI)			
TR	1.15^ǂ^	1.03, 1.29	p = 0.02*
Open TVR surgery	0.99^ǂ^	0.17, 5.65	p = 0.99
TTVR	1.14^ǂ^	0.48, 2.70	p = 0.77
Overall	1.16^ǂ^	1.06, 1.26	p = 0.0007*
New arrhythmia, RR (IV, random, 95% CI)			
TR	1.03^ǂ^	0.90, 1.17	p = 0.69
TTVR	0.26^ǂ^	0.03, 2.24	p = 0.22
Overall	1.02^ǂ^	0.90, 1.17	p = 0.75
New PPM implantation, RR (IV, random, 95% CI)			
TR	1.23^ǂ^	0.82, 1.84	p = 0.31
Open TVR surgery	1.57^ǂ^	1.21, 2.03	p = 0.0006*
TTVR	1.45^ǂ^	1.10, 1.93	p = 0.01*
Overall	1.46^ǂ^	1.23, 1.73	p < 0.0001*

Discussion

TR has shown a higher prevalence among females compared to males, but in-depth discussions regarding the pathophysiology, etiology, and outcomes of TR in both sexes are lacking. This systematic review and meta-analysis mark the first attempt to explore disparities in the characteristics and outcomes of males and females with TR, as well as those undergoing tricuspid valve repair. Our meta-analysis encompasses a significant sample of 22,574 patients from 12 studies, representing both sexes (47.5% males and 52.5% females), shedding light on sex-specific distinctions in TR's origins, pathophysiology, and clinical outcomes. Notably, sex-related variances in tricuspid valve disease epidemiology and ventricular responses to changes in loading conditions contribute to variations in TR disease occurrence and clinical outcomes among both sexes. Our meta-analysis underscores the undeniable importance of considering sex-based differences in TR's pathophysiology and management outcomes.

Demographic Characteristics

Our analysis of demographic characteristics has unveiled notable disparities between males and females. Females tend to present at an older age compared to males, hinting at age and female sex as potential determinants for TR, in alignment with the findings of a study conducted by Singh et al. [30]. The severity of TR, as gauged by tricuspid regurgitant volume, hinges on the difference between right ventricular stroke volume and main pulmonary artery forward flow [31]. Our analysis has shown that tricuspid regurgitant volume is higher in males than in females, suggesting a possibly greater disease severity in males. Still, such differences in volume can be attributed to differences in the body surface area (BSA) between both sexes. Additionally, males exhibited lower baseline LVEF and TAPSE when compared to females. These observations suggest that, despite a lower prevalence of TR in males, they may experience more severe TR than females. Future research is needed to explore the clinical value of such differences.

On the other hand, females exhibited higher PASP and systolic pulmonary arterial pressure (SPAP), both of which are linked with pulmonary hypertension. While our analysis has linked elevated PSAP to an increase in the severity of functional TR due to pulmonary hypertension, another study conducted by Multak et al. found that most individuals with elevated PSAP exhibited only mild TR [32]. Furthermore, the elevated PSAP observed among females may contribute to the higher incidence of functional TR, as observed by Gual-Capllonch et al. [21].

The study conducted by Gual-Capllonch et al. revealed that the independent predictors of significant functional TR (FTR) in females were identified as atrial fibrillation (AF), indexed tricuspid diameter annulus, and PASP [21]. This suggests that in female patients, these factors are key determinants of the development of significant FTR. Additionally, in a separate study by Ong et al., severe TR was investigated in a large echocardiogram database, and it was found in 1.2% of cases. Notably, the majority of these patients were female. They were classified into three distinct groups: (1) organic TR (11.3%), (2) functional TR (79.7%), primarily associated with conditions like pulmonary hypertension and left-sided heart disease, and (3) idiopathic TR (9%), which predominantly affected older individuals and those with AF. These findings shed light on the prevalence and underlying causes of severe TR in different patient groups, highlighting the multifactorial nature of this condition, particularly in females [33].

Ancona et al. reported a significantly higher mean value of the 3D diastolic annular area in males compared to females. The study's findings regarding sex or sex-related differences in tricuspid valve apparatus parameters showed that major and minor diastolic diameters (MDD and mDD) were significantly different between males and females. However, after normalization for BSA, the differences in normalized mDD and MDD between males and females were not significant. The tricuspid diastolic annular area exhibited significant differences between males and females, both in its raw form and after normalization for BSA. Tricuspid annular fractional area change did not show significant differences between males and females. These findings suggest that there are sex-related disparities in certain tricuspid valve parameters, but these disparities largely diminish when adjusted for body size [34].

In light of the study conducted by Afilalo et al., which investigated TR in patients with pulmonary hypertension and established the significant influence of tricuspid leaflet area (TLA) on TR severity [35]. Our findings revealed that the non-physiologic TR area was larger in the female group (2.71 ± 1.04 cm² vs. 4.22 ± 1.64 cm², p < 0.05), as demonstrated by Hui et al. Based on this, it can be postulated that females with a larger leaflet area are at a higher risk of developing TR. This inference underscores the potential clinical relevance of TLA in identifying patients, particularly females, who may benefit from interventions such as leaflet augmentation during tricuspid valve repair to improve outcomes in this patient population [23].

In our systematic review, Dietz et al. reported that females were more likely to have TR related to left valvular disease and isolated TR, while males were more prone to TR related to left ventricular function [20]. Regarding TR caused by endocarditis, Pfannmueller et al. found no sex difference in the source of endocarditis [25]. However, males with TR presented with endocarditis more frequently than females, as reported by Veen et al. [18]. In contrast, Saeed et al. discovered that females exhibited a higher prevalence of secondary TR than primary TR compared to males [28].

In several studies, it has been observed that females exhibit a higher prevalence of TR in specific contexts. Scotti et al. and Gual Capllonch et al. both found that females had a higher prevalence of left valvular disease-related TR compared to males (59% vs. 56% and 22.6% vs. 13.6%, respectively) [17,21]. Pfannmeuller et al. reported that TR due to previous cardiac surgery was more common in females (73.3%) than in males (42.9%) [25]. Furthermore, a study conducted by Song et al. revealed that the female sex was an independent risk factor for the development of significant TR following successful left-sided valve surgery, and females with late significant TR had lower clinical event-free survival rates compared to males (76% vs. 91%) [36]. These findings collectively suggest that females may have a higher risk of TR in specific cardiac conditions and post-surgical scenarios, potentially impacting their long-term clinical outcomes.

Sex-Specific Differences in Treatment Outcomes

Sex-specific differences in treatment outcomes after open heart surgery: In our analysis of treatment outcomes following open heart surgery, we observed no statistically significant difference between males and females in both in-hospital and late mortality. However, we noted a significant sex-based difference when considering the need for a new PPM, with males exhibiting an increased risk, as also observed in Khan et al. [24].

Sex-specific differences in treatment outcomes after TTVR:* *When analyzing treatment outcomes after TTVR, we found no statistically significant differences in in-hospital and late mortality between males and females, consistent with the results from Scotti et al. and Dietz et al. [17,20]. However, a statistically significant difference emerged in the need for a new PPM, with males at an increased risk, as also observed by Khan et al. [24]. Furthermore, we observed that males had a higher risk of developing arrhythmias following TTVR; however, these results were statistically insignificant [27,28].

Overall sex-specific differences irrespective of treatment option or no treatment: Considering in-hospital mortality following either open surgery or TTVR, our analysis indicated no statistically significant differences between males and females. Nevertheless, we noted a statistically significant sex-based difference in terms of late mortality, with males exhibiting a slightly higher risk than females, irrespective of the type of procedure or no procedure. Additionally, we found a significant difference between males and females regarding the need for a new PPM, with males at an increased risk. In terms of developing arrhythmias, males had an overall higher risk, regardless of any intervention or lack thereof; however, these results were statistically insignificant [27,28].

These findings collectively emphasize the importance of considering sex-specific differences in TR patients when making clinical decisions. The differences in baseline characteristics and outcomes between males and females suggest that personalized treatment strategies may be warranted. Additionally, further research is needed to explore the underlying mechanisms contributing to these disparities, as this can inform better management and outcomes for both male and female TR patients. Overall, our study underscores the significance of accounting for sex-based disparities in TR management and treatment outcomes, which can ultimately lead to improved patient care.

Limitations

The study's limitations include the heterogeneity of the included studies, potential publication bias, and a lack of comprehensive demographic and clinical data. Historical studies, variations in treatment timing, and a retrospective design also present challenges. Furthermore, the limited number of studies warrants caution when generalizing the conclusions of our study. Ethnic and racial diversity among patients was not extensively addressed. These limitations underline the need for more focused and prospective research in this area.

Future directions

Considering the current findings, future research endeavors should delve deeper into understanding the physiological and molecular underpinnings contributing to sex-based disparities in TR. This knowledge will enable the development of personalized treatment approaches, considering patient demographics and clinical factors. Investigating potential sex-specific biomarkers for early diagnosis and risk assessment can improve patient care, as can patient-centered studies assessing the long-term quality of life and health disparities experienced by male and female TR patients. Educational initiatives for healthcare professionals, collaboration across multiple healthcare institutions, and well-designed clinical trials that consider sex-specific outcomes are crucial for advancing the field, enhancing treatment efficacy, and ensuring equitable care for all patients.

## Conclusions

Our comprehensive meta-analysis sheds light on sex-based disparities in TR, revealing distinct baseline characteristics and treatment outcomes between male and female patients. While males generally exhibited higher baseline cardiac function, they required more PPM across various treatment options. There was no difference in terms of short-term mortality. However, late survival was better in female patients.
